# Systematic Multiomic Analysis of *Ly75* Gene Expression and Its Prognostic Value through the Infiltration of Natural Killer (NK) Cells in Skin Cutaneous Melanoma

**DOI:** 10.3390/jcm9051383

**Published:** 2020-05-08

**Authors:** Minchan Gil, Kyung Eun Kim

**Affiliations:** 1 Department of Stem Cell and Regenerative Biotechnology, Konkuk University, Seoul 05029, Korea; minchangil@gmail.com; 2 Department of Cosmetic Sciences, Sookmyung Women’s University, Seoul 04310, Korea; 3 Nano-Bio Resources Center, Sookmyung Women’s University, Seoul 04310, Korea

**Keywords:** *Ly75*, CD205, skin cutaneous melanoma (SKCM), prognostic factor, natural killer (NK) cells, immune cell infiltration

## Abstract

*Ly75* (also known as DEC-205 or CD205) is expressed in immune cells and cancers and involved in tumor immunity. However, clinical relevance of *Ly75* expression in skin cutaneous melanoma (SKCM) have not been comprehensively studied. This study analyzed the correlation between *Ly75* mRNA expression and patient survival using systematic multiomic analysis tools. *Ly75* mRNA expression level was significantly lower in SKCM tissues than in normal tissues. Survival analysis showed that *Ly75* expression significantly correlated with good patient survival. To determine possible mechanisms, the association between *Ly75* expression and immune cell infiltration was analyzed. *Ly75* expression was positively correlated with various infiltrated immune cells, particularly with natural killer (NK) cell infiltration and activation in SKCM. Moreover, analysis of *Ly75*-co-altered gene expression revealed that *Ptprc* (CD45) was most significantly correlated with *Ly75*. Gene ontology analysis of *Ly75*-co-altered genes indicated the relation to lymphocyte activation, including NK cell activation. Overall, our study provides the first clinical evidence that *Ly75* expression is significantly associated with melanoma patient survival and NK cell infiltration, suggesting that *Ly75* could be a useful prognostic factor.

## 1. Introduction

Melanoma is the most malignant skin cancer with tremendously poor patient prognosis, and is responsible for the highest mortality rate among skin cancers [1]. In the United States, melanoma is currently known as the fifth most common cancer (https://seer.cancer.gov/statfacts/html/melan.html). Owing to its highly metastatic properties, there have recently been tremendous efforts towards early detection and primary prevention of melanoma. However, despite such efforts, as of 2018, the incidence has been increasing at a much faster rate than that of other types of cancer [2,3]. Additionally, melanoma is known to be a highly resistant cancer to chemotherapy, owing to its resistance to apoptosis [4]. As such, several therapeutic approaches have been developed; however, no effective therapies have yet emerged. Many researchers have previously explored using immunotherapy to regulate the immune response of the host. Recently, using cancer immunotherapy to regulate the immune responses involved in melanoma has received attention, as melanoma is known as one of the most highly immunogenic tumors that respond to immunological manipulations—such as the inhibition of immune checkpoints—in a tumor microenvironment (TME) [5]. Moreover, tumor-infiltrating lymphocytes (TILs) comprise numerous effector cells, namely cluster of differentiation (CD)8^+^ T cells and natural killer (NK) cells, in a tumor microenvironment, and effectively act as a prognostic factor in melanoma patients. Recently, as the importance of TILs in TME has been emphasized, studies on immune responses by TIL in TME have increased. Indeed, many studies have reported that the distribution of TILs in melanoma patients leads to a better prognosis, thus, TILs are increasingly gaining attention for their implications in melanoma treatment and predicting prognosis [6,7,8]. However, since TILs includes not only effector cells having a strong anti-cancer effect but also immunosuppressive cells as a heterogenous group, a distribution of many immunosuppressive cells can rather inhibit the effector cell activities [9,10,11]. That is, there is currently no conclusive evidence that TILs act as a strong prognostic factor in melanoma. Therefore, studies of the phenotype and function of TILs in melanoma are needed for predicting prognosis. In particular, there are many studies to identify novel biomarkers that can predict the infiltration of effector cells, such as NK cells and CD8^+^ T cells [12,13,14,15].

NK cells are innate lymphoid cells (ILCs) that are characterized as CD56^+^CD3^−^ cytotoxic lymphocytes. NK cells are generally divided into two populations, namely CD56^bright^ and CD56^dim^ subpopulations, which play critical roles in the innate immune system via the cytotoxic activity of the CD56^dim^ subpopulation and the cytokine production of the CD56^bright^ subpopulation [16,17]. NK cell activation is mediated by the balance between activating and inhibitory receptors. [18]. Activated NK cells induce the activation of a caspase cascade and ultimately, apoptosis occurs in tumor cells via the stimulation of Fas receptor on tumor cells interacted with Fas ligand on NK cells and the release of granules, such as granzymes and perforin from NK cell into the tumor cells [19,20]. Generally, the activity and functions of NK cells are reported to be significantly suppressed in the patients with melanoma, leading to tumor immune escape [21]. However, a recent study has demonstrated that CD56^dim^ NK cells are generated and recruited in tumor-infiltrated lymph nodes in patients with melanoma [22]. These cells have the characteristic of expressing CD57^dim^CD69^+^C-C chemokine receptor type (CCR)7^+^Killer-cell immunoglobulin-like receptor (KIR)^+^ on the surface of the cell and have been shown to exhibit strong cytotoxicity against autologous melanoma cells, suggesting that the cytolytic activity of NK cells may not be inhibited in patients with melanoma as has been reported [22].

Ly75, a type I transmembrane surface protein, is a member of the mannose receptor family, including M-type phospholipase A2 receptor (PLA2R) and The Urokinase Receptor Associated Protein (uPARAP/Endo180) [23]. *Ly75* is also known as CD205 or DEC-205, and is expressed by various immune cells, including T cells, B cells, monocytes, and NK cells [24,25]. In particular, *Ly75* is predominantly expressed by dendritic cells (DCs), and plays a critical role in endocytosis and antigen presentation to T cells through major histocompatibility complex (MHC) molecules, thereby resulting in anti-tumor responses [26,27]. It has also been reported that the ablation of *Ly75*-expressing DCs does not generate cytotoxic T lymphocytes in vivo, indicating that *Ly75*-expressing DCs effectively generate cytotoxic T lymphocytes to kill infected pathogens [28]. In addition, an in vivo study shows that the depletion of *Ly75*-expressing DCs impairs tumor progression in ovarian cancer [29]. Therefore, these studies suggest that *Ly75* have an important role in the anti-tumor responses.

Although the roles of *Ly75* in tumors have been reported both in vivo and in vitro, there has been no comprehensive analysis on the clinical relevance of *Ly75* expression in skin cutaneous melanoma (SKCM). Therefore, this study systematically investigated *Ly75* mRNA expression and its correlation with cancer prognosis in melanoma patients. Moreover, to identify related factors that affect survival rates, we also investigated the correlation between *Ly75* expression and tumor-infiltrating lymphocytes, especially NK cells, in the tumor microenvironment. In conclusion, this study provides evidence for the potential of using *Ly75* expression as a prognostic marker for melanoma and its correlation with the infiltration and activation of NK cells.

## 2. Experimental Section

### 2.1. Ly75 mRNA Expression and Genome Alteration in Cancers

*Ly75* expression in various cancers were compared to their normal counterparts in various types of cancer using the Gene Expression Profiling Analysis (GEPIA) tool (http://gepia.cancer-pku.cn/) [30], Oncomine database version 4.5 (Thermo Fisher Scientific Inc., Ann Arbor, MI, USA) (https://www.oncomine.org/resource/login.html) [31] and Gene Expression Across Normal and Tumor Tissue 2 (GENT2) databases (http://gent2.appex.kr/gent2/) [32,33]. GEPIA offers analysis tools for gene expression data of The Cancer Genome Atlas (TCGA) of tumor samples and their normal controls composed of paired adjacent TCGA normal tissue and Genotype-Tissue Expression (GTEx) normal tissue, which are recomputed on a uniform bioinformatic pipeline to eliminate batch effects in the University of California, Santa Cruz (UCSC) Xena Project [34]. The tissue source of normal controls and sample numbers of datasets used in GEPIA were detailed in Supplementary Table S1. The Oncomine analysis provides comprehensive analytical tools on multiple microarray datasets of cancer transcriptome. The GENT2 offers microarray-based gene expression profiles across various types of cancers and their normal tissues in the Affymetrix U133plus2 or U133A platforms using collected data from public resources. All queries were performed with defaults settings in GEPIA and GENT2. *Ly75* expression in various cancers were also explored using the Oncomine database with a threshold *p*-value of < 10^−4^, fold change of > 2, and all gene ranking (https://www.oncomine.org/resource/login.html) [31,35]. *Ly75* expression in melanoma and normal skin was retrieved from the TCGA TARGET GTEx cohort in the UCSC Xena Browser (http://xena.ucsc.edu/). UALCAN web (http://ualcan.path.uab.edu/index.html) was used for the analysis of the promoter methylation of *Ly75* from the TCGA-skin cutaneous melanoma (SKCM) dataset tool [36]. The cBioPortal database version 3.2.14 (http://www.cbioportal.org/) was utilized to analyze mutations and conduct copy number alteration (CNA) analyses on the TGCA PanCanAtlas datasets using default parameter settings [37,38]. Correlation of *Ly75* expression with each alteration status was plotted. An unpaired t-test was used for statistical analysis in the GraphPad 7 software (GraphPad software, San Diego, CA, USA).

### 2.2. Prognostic Value of Ly75 Expression in Various Tumors

Prognostic value of *Ly75* mRNA expression was first examined across TCGA datasets using the OncoLnc (http://www.oncolnc.org/) online analysis tool [39] and subsequently using GEPIA. Patient samples were split into two groups with the median values of *Ly75* expression and analyzed using both Kaplan–Meier survival curves and the log-rank test in GEPIA. The Kaplan–Meier Scanner module in R2: Genomics Analysis and Visualization Platform (https://hgserver1.amc.nl/cgi-bin/r2/main.cgi) was utilized to generate survival curves comparing the two patient groups that were split by *Ly75* expression levels, which was chosen to minimize the log–rank *p*-value with set of minimal group size to 10% of sample numbers in each subgroup. The survival analysis was done with the SKCM–TCGA dataset (*n* = 470), its subgroups of gender, age, and tumor stage (only in stage i, ii, iii, and iv), and dataset GSE19234.

### 2.3. Analysis of the Association of Ly75 Expression with Immune Infiltration

The correlation between *Ly75* expression and tumor-infiltrating immune cells in the TCGA datasets was examined using the Tumor Immune Estimation Resource (TIMER) web version 1 (https://cistrome.shinyapps.io/timer/) [29]. The correlation values of *Ly75* expression levels with tumor purity and the abundance of various types of immune cells were retrieved for each tumor. The correlation between *Ly75* and the genetic signatures of immune cells was analyzed with GEPIA. The genetic signatures of each type of immune cells were used as previously described [13,14]. The correlation of *Ly75* expression with the genetic signatures of activated NK cells were analyzed with the Spearman’s correlation in correlation modules of the TIMER2.0 web tool (http://timer.cistrome.org/).

### 2.4. Profiling and Ontology Analysis of Co-Expressed Genes with Ly75

The co-expression genes of *Ly75* were examined using the TCGA–SKCM dataset with cBioportal. Next, 24 of the strongest correlated genes with the highest Spearman’s correlation coefficient values and lowest *p*-values were retrieved. The correlation between *Ly75* and the top positively correlated gene was further confirmed by a heatmap and scatter plot using the TCGA–SKCM dataset through the UCSC Xena Browser. The correlation between *Ly75* and the top positively correlated gene was also identified using the Tumor Melanoma Metastatic- Bhardwaj- 44 dataset with the R2: Genomics Analysis and Visualization Platform. To identify gene ontology terms that were shared by *Ly75* and its correlated genes, we utilized the Enricher (https://amp.pharm.mssm.edu/Enrichr) [40]. The enriched gene ontology terms were expressed as bar diagrams.

### 2.5. Reproducibility of Data Presented from Web Tools

All of the analytical results from web tools were reconfirm or updated at 20 April, 2020. 

## 3. Results

### 3.1. Ly75 mRNA Expression in Various Types of Cancers

**Figure 1:**
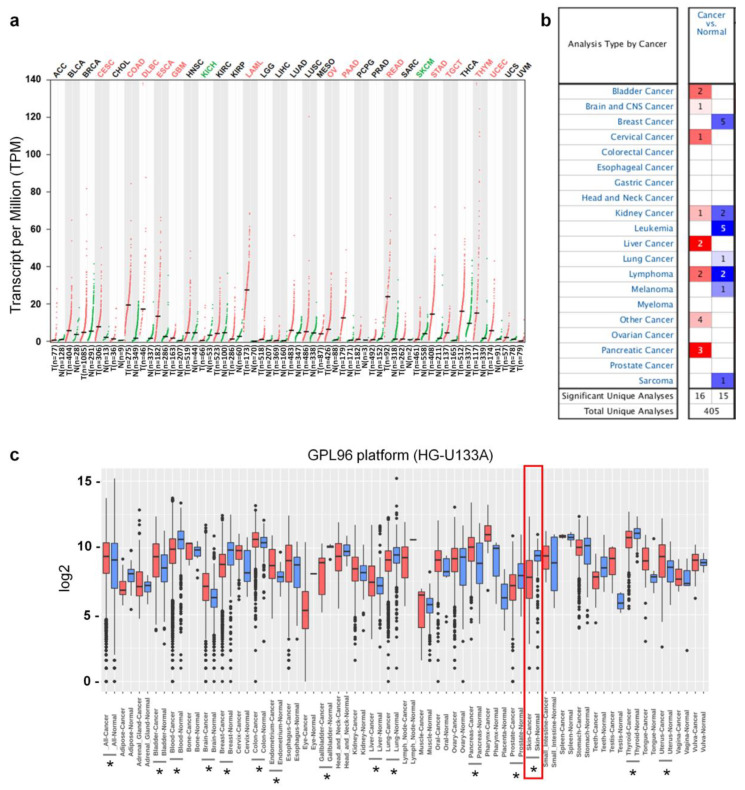
Transcription levels of *Ly75* in different cancer type and their normal tissues. (**a**) *Ly75* mRNA expression levels in various cancer type and their normal counterparts were retrieved from the Gene Expression Profiling Interactive Analysis (GEPIA) web tool (http://gepia.cancer-pku.cn/) as dot plots. Abbreviations of the names of cancer types and number of samples are listed in Supplementary Table S1. Cancer types in which *Ly75* expression was significantly changed compared to normal counterparts were marked as red (increase) or green (decrease). (**b**) The number of datasets with *Ly75* increased mRNA expression (left column, red) and decreased-expression (right column, blue) in cancers compared to normal tissues was retrieved using the Oncomine database (https://www.oncomine.org/) with the parameters: *p*-value of 10^−4^, fold change of 2, and all gene ranking. (**c**) Boxplot for the *Ly75* expression profile across cancer type generated with the Gene Expression Across Normal and Tumor Tissues (GENT2) database (http://gent2.appex.kr/gent2/). The statistical significance of the difference between each type of cancer and its corresponding normal tissue was estimated using a two-sample T-test, and *p*-values of less than 0.01 are marked by asterisks.

It has been reported that *Ly75*, which is expressed in various immune cells, such as dendritic cells (DCs), T cells, and natural killer cells (NK cells), plays an important role in antigen presentation and the initiation of immune responses [24,25]. Although *Ly75* expression also regulates tumor metastasis in various types of cancer, including ovarian cancer [29,41], the roles and clinical significance of *Ly75* in melanoma have not yet been studied. To compare the mRNA expression levels of *Ly75* in tumor and normal tissues, differences in the *Ly75* mRNA levels between various tumor tissues and normal control were analyzed using the RNA-seq data from the TCGA and GTEx datasets. Figure 1a shows that *Ly75* mRNA expression levels were significantly lower in skin cutaneous melanoma (SKCM) and kidney chromophobe (KICH) compared to in normal tissues. However, *Ly75* mRNA expression levels were elevated in cervical squamous cell carcinoma and endocervical adenocarcinoma (CESC), colon adenocarcinoma (COAD), lymphoid neoplasm diffuse large B-cell lymphoma (DLBC), esophageal carcinoma (ESCA), glioblastoma (GBM), acute myeloid leukemia (LAML), ovarian serous cystadenocarcinoma (OV), pancreatic adenocarcinoma (PAAD), rectum adenocarcinoma (READ), stomach adenocarcinoma (STAD), testicular germ cell tumors (TGCT), thymoma (THYM), and uterine corpus endometrial carcinoma (UCEC). *Ly75* expression levels in tumor and normal tissues were further analyzed using the Oncomine database, as shown in Figure 1b. The number of datasets indicated both the overexpression (red) and underexpression (blue) of *Ly75* mRNA in tumor versus normal tissues. These data were obtained with the parameters of a *p*-value of 10^−4^, fold change of 2, and gene ranking of all. The comparison of *Ly75* mRNA expression levels between tumor and normal tissues also revealed downregulation in melanoma. Additionally, as shown in Figure 1c, it was confirmed using the GENT2 database that *Ly75* mRNA expression was lower in skin cancer. Collectively, systematic analysis using various databases shows that *Ly75* mRNA expression in melanoma is lower than in normal tissues, suggesting lowered expression of *Ly75* may be involved in the progression of melanoma.

### 3.2. Prognostic Value of Ly75 mRNA Expression in SKCM

**Figure 2:**
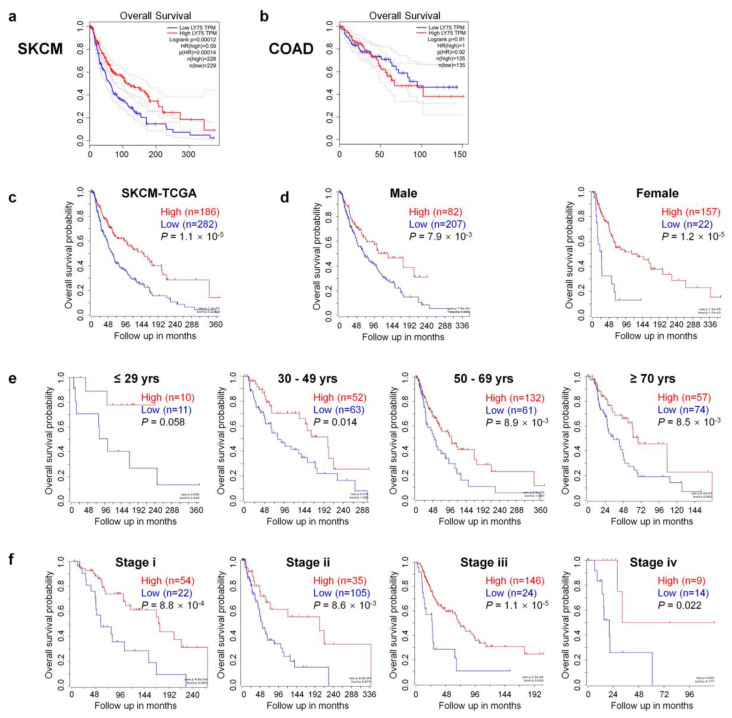
Comparison of overall survival between *Ly75* expression high and low patient groups in skin cutaneous melanoma (SKCM) and colon adenocarcinoma (COAD). Kaplan–Meier survival curves of two patient groups that were split by the median value of *Ly75* expression were generated from The Cancer Genome Atlas (TCGA) data using the Gene Expression Profiling Analysis (GEPIA) web tool for (**a**) skin cutaneous melanoma (SKCM) and (**b**) colon adenocarcinoma (COAD). The log-rank *p*-value, Cox proportional hazard ratio (HR), *p*-value of HR, and number of patients in each analysis group were shown on graph. Moreover, 95% confidence interval was marked with dotted lines. Kaplan–Meier survival curves of two patient groups that were split by *Ly75* expression with the scan-cut-off-modus were scanned by R2: Genomics Analysis and Visualization Platform (https://hgserver1.amc.nl/cgi-bin/r2/main.cgi) with the (**c**) SKCM-TCGA dataset (*n* = 470) and its subgroups divided by (**d**) gender, (**e**) age, and (**f**) tumor stage (only in stage i, ii, iii, iv).

To investigate the association between *Ly75* mRNA expression and patient prognosis in various cancer types, overall survival rates were compared using the Cox regression model and the OncoLnc online tool. Positive and negative cox regression results for *Ly75* expression are shown in Supplementary Table S2. Three cancer types including SKCM, sarcoma (SARC), and low grade glioma (LGG) showed significant COX regressions for *Ly75* mRNA expression (*p* < 0.01). In SKCM and SARC, a significant positive correlation was found between *Ly75* expression and patient survival, whereas in LGG, a negative correlation was observed. No significant correlation was found between *Ly75* expression and patient survival in most cancer types, including colon adenocarcinoma (COAD). Furthermore, COAD was used as a negative control in subsequent analyses. In addition to COX regression results for *Ly75* in OncoLnc, overall survival by *Ly75* expression levels was visualized using Kaplan–Meier survival curves in SKCM and COAD. As shown in Figure 2a,c, overall survival was significantly positively correlated with *Ly75* expression in patients with SKCM. With COAD as a negative control, no correlation was found between *Ly75* expression and overall survival (Figure 2b). Additionally, the correlation between *Ly75* mRNA expression and overall survival according to various clinicopathological parameters, such as gender, age, and stage, was analyzed in SKCM (Figure 2d–f). Significant correlations were found between *Ly75* expression and overall survival in most clinicopathological parameters, except for tumor stage 1. Overall, these results suggest that *Ly75* mRNA expression fosters different prognostic effects based on cancer type. In SKCM, *Ly75* mRNA expression exhibited a significant positive correlation with patient survival, therefore, we suggest the prognostic value of *Ly75* mRNA expression for predicting the overall survival of patients with SKCM.

### 3.3. Ly75 Gene Expression and Genome Alterations in SKCM

**Figure 3:**
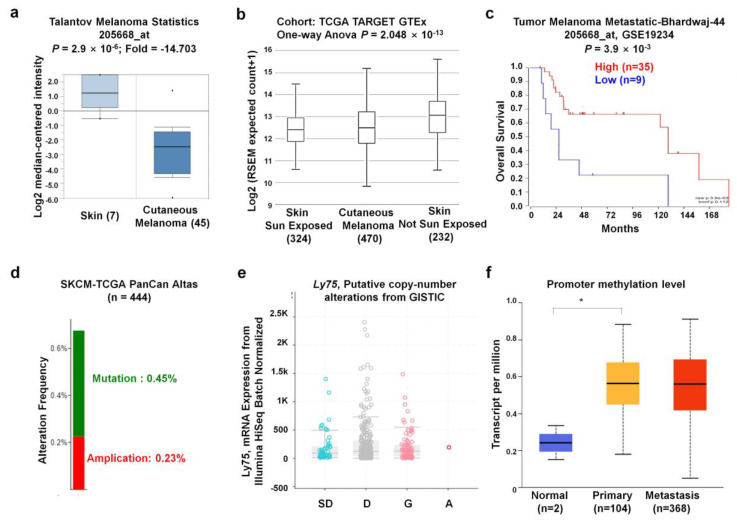
*Ly75* expression and genome alterations in SKCM. (**a**) The boxplot of *Ly75* expression in cancer tissues (right plot, *n* = 45) and normal (left plot, *n* = 7) was retrieved from the Talantoy melanoma dataset in the Oncomine database. (**b**) *Ly75* expression in normal skin of sun-exposed (Lower Leg) and non-sun-exposed (Suprapubic) skin, and melanoma datasets from TCGA, TARGET, and Genotype-Tissue Expression (GTEx) in the University of California, Santa Cruz (UCSC) Xena browser (http://xena.ucsc.edu/). (**c**) The Kaplan–Meier survival curves of the two patient groups of Tumor Melanoma Metastatic-Bhardwaj-44 dataset split by *Ly75* expression with “scan” option. A group has higher *Ly75* expression denoted by red color and lower expression by blue color using R2: platform (https://hgserver1.amc.nl/cgi-bin/r2/main.cgi). (**d**) The mutation and alteration frequencies of the *Ly75* gene were determined from the SKCM (TCGA, PanCan atlas) (*n* = 444) dataset using the cBioPortal database (https://www.cbioportal.org/). (**e**) Correlation of *Ly75* mRNA expression and copy number alteration status in terms of shallow deletion (SD), diploid (D), gain (G), and amplification (A). (**f**) Promoter methylation of *Ly75* was examined from the TCGA-SKCM dataset through the UALCAN web tool (http://ualcan.path.uab.edu/index.html).

To further determine the lower expression of *Ly75* mRNA in SKCM, we analyzed other datasets in the Oncomine database. The Talantoy melanoma datasets suggest that *Ly75* expression was significantly downregulated in cutaneous melanoma tissues compared to normal tissues, as observed in Figure 3a. Moreover, *Ly75* expression in melanoma and normal skins, including sun-exposed and non-sun-exposed skin, was analyzed using datasets from TCGA TARGET GTEx in the UCSC Xena Browser. Interestingly, Figure 3b shows that *Ly75* expression was downregulated in cutaneous melanoma compared to non-sun-exposed skin, but not compared to sun-exposed skin, implicating that *Ly75* expression may be affected by sun exposure. Similar to Figure 2a,c, also showed that overall survival rate of patients with high *Ly75* expression (*n* = 35) was significantly higher compared with patients with lower expression (*n* = 9), as based on the Tumor Melanoma Metastatic-Bhardwaj-44 dataset. Therefore, these results suggest that *Ly75* expression is significantly lower in SKCM tissues than in normal skin without sun exposure and exhibits positive correlation with patient survival. 

Next, gene alterations of the *Ly75* gene in the TCGA PanCan Atlas dataset were analyzed using the cBioPortal website. The mutation and alteration frequencies of the *Ly75* gene were 0.45% and 0.32%, respectively (Figure 3d). Gene expression was not significantly altered by the copy number alteration status. The relatively low alteration rate and the lack of relevance of expression with the copy number alteration status implicate that *Ly75* expression was not due to changes in chromosome alteration. Promoter methylation is an epigenetic regulatory factor, and abnormal methylation is evident in tumors [42]. To examine the methylation status of the *Ly75* gene in SKCM, the TCGA-SKCM dataset through the UALCAN web tool was used. As observed in Figure 3f, promoter methylation was markedly increased in primary and metastasis melanoma compared to in normal tissues, suggesting the presence of a significantly negative correlation between mRNA expression and DNA methylation of *Ly75*.

### 3.4. Immune Cell Infiltration by Ly75 mRNA Expression

**Figure 4:**
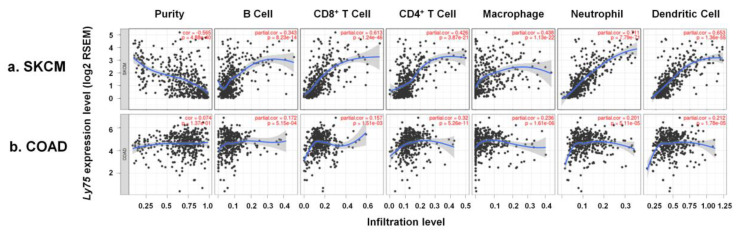
Association of *Ly75* expression with immune cell infiltration in SKCM and COAD. Scatter plots were generated using with the Tumor Immune Estimation Resource (TIMER) web tool (https://cistrome.shinyapps.io/timer/) to identify the immune cell profiles that are associated with *Ly75* expression in (**a**) SKCM and (**b**) COAD of the TCGA database. Correlation constants and *p*-values are detailed in Supplementary Table S3.

Tumor microenvironment (TME) is composed of various types of cell, including immune cells and stromal cells as well as cancer cells. These cells actively interact with each other to induce tumor immune escape, leading to tumor metastasis [43]. Moreover, several studies have reported that the molecular composition in the TME exhibits a critical role for tumor progression and immune responses [44,45,46]. Importantly, levels of immune cell infiltration are known as a prognostic factor in patients with melanoma [47]. Therefore, in this study, the correlation between *Ly75* expression levels and immune cell infiltration was investigated to determine the mechanisms related to increased patient survival by high *Ly75* mRNA expression. As shown in Figure 4a, analysis of the data by TIMER shows that *Ly75* expression had a significantly negative correlation with tumor purity in SKCM (cor. = −0.565, *p* = 4.88 × 10^−40^), indicating that *Ly75* in SKCM may be expressed by infiltrated immune cells [48]. Moreover, a significant positive correlation was found between *Ly75* expression levels and immune cell infiltration, such as B cells (cor. = 0.343, *p* = 8.23 × 10^−14^), CD8^+^ T cells (cor. = 0.613, *p* = 1.24 × 10^−46^), CD4^+^ T cells (cor. = 0.426, *p* = 3.87 × 10^−21^), macrophages (cor. = 0.438, *p* = 1.13 × 10^−22^), neutrophils (cor. = 0.711, *p* = 7.79 × 10^−71^), and dendritic cells (cor. = 0.653, *p* = 1.36 × 10^−55^). *Ly75* was reported to be expressed by various immune cells, including dendritic cells (DCs), T cells, B cells, monocytes, and macrophages. Therefore, *Ly75* is mainly expressed in infiltrating immune cells in the tumor environment of SKCM. In contrast, Figure 4b shows that *Ly75* expression was not correlated with tumor purity or immune infiltration in COAD.

In addition, to examine the relationship between *Ly75* expression and the infiltration of various immune cell subsets in more detail, the correlation between *Ly75* expression and gene marker expression of each immune cell was examined in SKCM. Table 1 includes various subsets of T cells (general T cells, CD8^+^ T cells, Th1 cells, Th2 cells, follicular Th cells, Th17 cells, γδ T cells, regulatory T cells), B cells, monocytes, tumor-associated macrophages (TAM), M1 macrophages, M2 macrophages, neutrophils, NK cells, and dendritic cells (DCs) in SKCM. As shown in Table 1, the mRNA expression of most immune cell markers was positively correlated with *Ly75* mRNA expression in SKCM, whereas *Ly75* expression was not significantly correlated with the expression of gene markers in COAD, which was used as a negative control. In particular, CD8^+^ T cells, NK cells, and γδ T cells are known to act as effector cells against tumors, allowing the enhancement of immune responses in the tumor microenvironment. This study shows that the infiltration of these anti-tumor effector cells was significantly increased by *Ly75* expression (Table 1). This was also confirmed using the GEPIA database, as shown in Table 2. A significant positive correlation was exhibited between *Ly75* expression and gene markers for CD8^+^ T cells, NK cells, and γδ T cells in SKCM, but not in COAD. Moreover, no correlation was found between *Ly75* expression and gene marker expression in the normal tissues of SKCM. Overall, these data suggest that higher *Ly75* expression regulates the infiltration of anti-tumor effector cells in SKCM, thereby enabling patient survival through their related cytotoxic activities.

### 3.5. Association between Ly75 Expression and NK Cell Activation in SKCM

**Figure 5:**
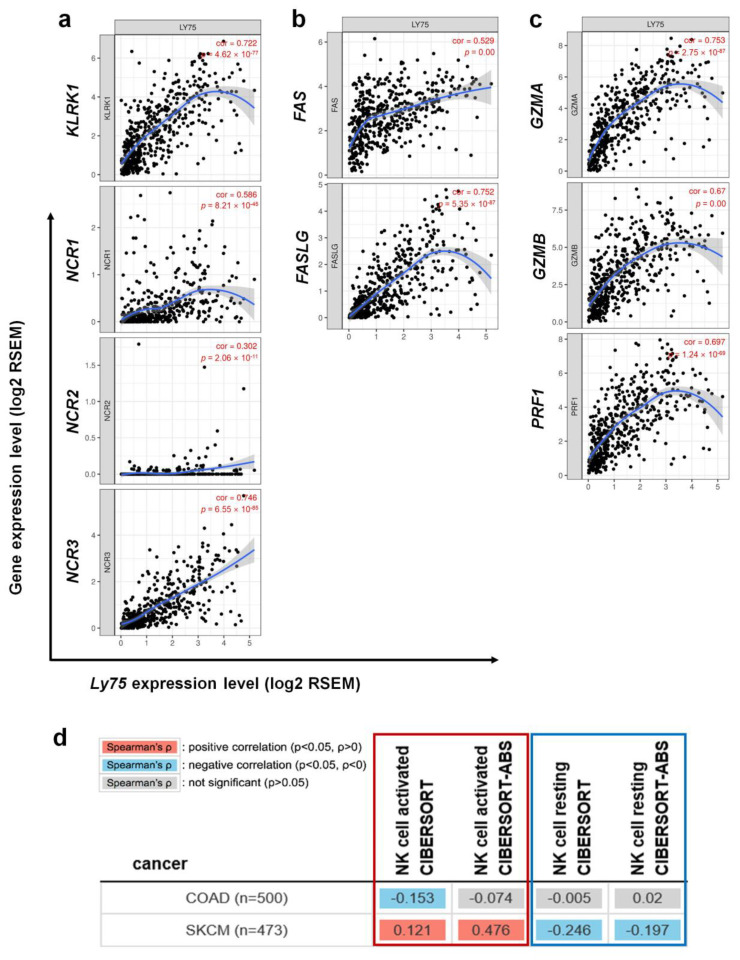
Correlation of *Ly75* with signature genes of NK cell cytolytic activity in SKCM. Expression correlations of *Ly75* and genes related to NK cell activation were explored with TIMER. (**a**) Activation receptors of activated NK cells, (**b**) FAS and the FAS ligand, and (**c**) Cytotoxicity signature genes. (**d**) Association of *Ly75* expression with infiltration of activated NK cells (red box) or resting NK cells (blue box) was analyzed using TIMER2.0 web tool (http://timer.cistrome.org/). Gene signatures of activated NK cells and resting NK cells from CIBERSORT were used on the web tool.

NK cells in patients with malignant melanoma are known to be impaired, rendering these cells unable to induce effective anti-tumor responses [21]. However, a recent study has reported that CD56^dim^ NK cells are generated and recruited in the tumor-infiltrated lymph node of melanoma patients [22], indicating that the cytolytic activity of NK cells is critical for anti-tumor responses in melanoma. Although *Ly75* is known to be expressed in NK cells and immune cells, the relationship between *Ly75* expression and NK cell activation has not yet been studied. This study investigated whether *Ly75* expression affects NK cell activation by determining the correlation between *Ly75* expression and gene expression of NK cell surface molecules, which are related to cytolytic activity, using the TIMER tool. As shown in Figure 5a,b, and Table 3, expression of *KLRK1* (NKG2D), *NCR1* (NKp46), *NCR2* (NKp44), *NCR3* (NKp30), and *FASLG* (Fas ligand) was considerably increased by *Ly75* expression in SKCM. Moreover, NKG2D and NCRs are known to act as activating receptors on NK cells, thus resulting in the activation of NK cells by interactions with corresponding ligands [49,50]. Fas ligand on NK cells interacts with the Fas receptor on tumor target cells, inducing the apoptosis of target cells [51]. The gene expression of these surface molecules ultimately exhibited a positive correlation with *Ly75* expression, suggesting that the increased expression of *Ly75* could regulate NK cell activation in SKCM. In addition, cytolytic molecules, such as granzymes and perforin, play critical roles in the anti-tumor responses of NK cells [51]. Perforin binds to the surface of target tumor cells, generating pores, and then granzymes, particularly granzyme A and B, enter the target cells through pores and stimulate apoptotic cascades. Figure 5c and Table 3 show that high *Ly75* expression was positively correlated with increased gene expression of granzyme A, granzyme B, and perforin in SKCM. However, no correlation was found between *Ly75* expression and all gene expression in COAD (Table 3). To further confirm the correlation between *Ly75* expression and NK cell activation, TIMER2.0 web tool was used. As shown in Figure 5d, *Ly75* expression was positively correlated with the infiltration of activated NK cells, but had the opposite effect with that of resting NK cells. This suggests that *Ly75* expression in SKCM tissue predicts the infiltration of specially activated NK cells. Overall, these findings indicate that *Ly75* expression regulates NK cell infiltration and cytolytic activity in SKCM, leading to improved prognosis in patients with melanoma.

### 3.6. Co-Expressed Genes with Ly75 in SKCM

**Figure 6:**
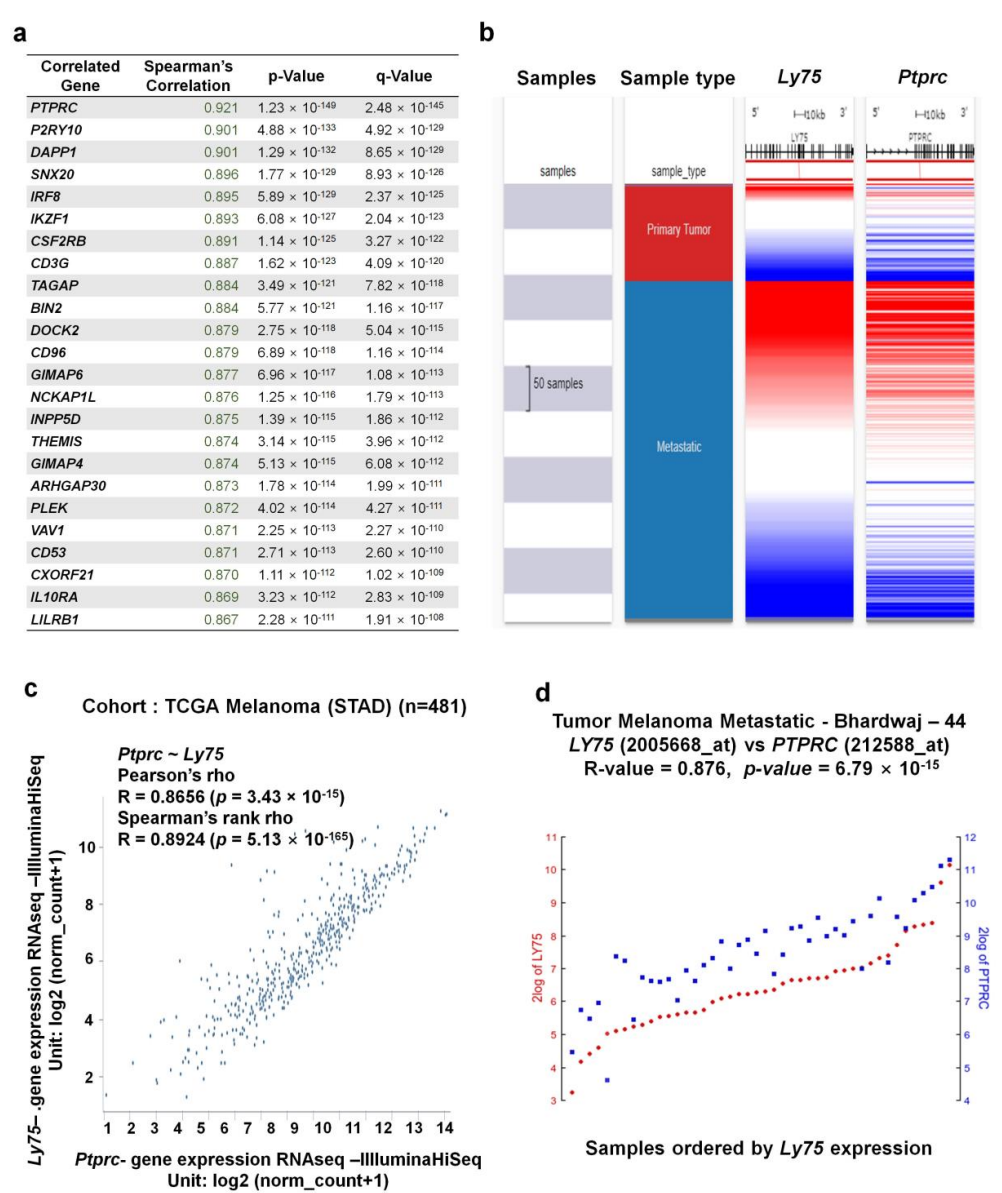
Co-expression genes of the *Ly75* gene in SKCM. (**a**) The 24 genes that are the most co-expressed with *Ly75* were analyzed using the TCGA-SKCM dataset and cBioportal (**b**) A heatmap of *Ly75* and *Ptprc* mRNA expression in the TCGA-SKCM dataset, determined using the UCSC Xena Browser. (**c**) Correlation between *Ly75* and *Ptprc* mRNA expression in the TCGA-SKCM dataset, as determined using the UCSC Xena Browser. (**d**) Correlation between *Ly75* and *Ptprc* mRNA expression in the Tumor Melanoma Metastatic - Bhardwaj - 44 dataset, as determined using the R2 platform.

Next, we investigated genes that exhibit correlated expression with *Ly75* in melanoma using the SKCM-TCGA dataset and cBioportal. We identified 24 genes that are the most positively co-expressed with *Ly75* in melanoma (Figure 6a). The gene expression of *protein tyrosine phosphatase receptor type C (Ptprc)* exhibited the strongest positive correlation with *Ly75* in the TCGA-SKCM dataset (R = 0.921). We also analyzed the co-expression patterns of *Ly75* and *Ptprc* of the TCGA-SKCM dataset with a heatmap (Figure 6b) and a dot plot (Figure 6c) using the UCSC Xena web tool. Co-expression patterns of *Ly75* and *Ptprc* in primary and metastatic melanoma were visualized using a heatmap (Figure 6b). Lastly, we confirmed this strong positive correlation between *Ly75* and *Ptprc* expression using Pearson (R = 0.8656) and Spearman (R = 0.8924) correlation analyses (Figure 6c). Strongly associated *Ly75* and *Ptprc* expression was also confirmed using the Tumor Melanoma Metastatic-Bhardwaj–44 set with the R2 platform (R = 0.876, *p* = 6.79 × 10^−15^), as shown in Figure 6d.

**Figure 7:**
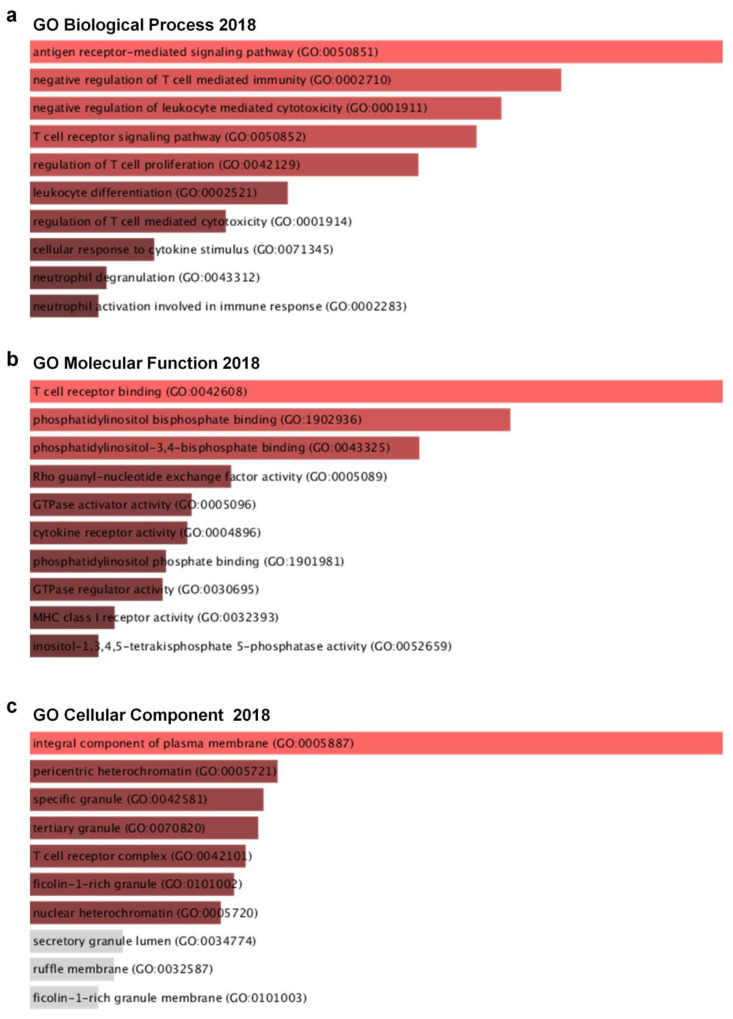
*Ly75* gene and its co-expressed genes are involved in signaling pathways in SKCM: Gene ontology analysis were carried out with *Ly75* gene and its top 25 co-expressed genes with the Enricher web tool (https://amp.pharm.mssm.edu/Enrichr). (**a**) Enrichment of GO Biological Process (2018), (**b**) Enrichment of GO Molecular Function (2018), and (**c**) Enrichment of GO Cellular Component (2018).

To identify possible biological processes and functions that are associated with *Ly75* and its co-altered genes in melanoma, we performed ontology analysis with *Ly75* and its 24 co-altered genes, which are shown in Figure 7. Using gene ontology (GO) biological process analysis, *Ly75* and its 24 positively co-altered genes were mainly related to T cell- and neutrophil-associated biological processes (Figure 7a). Using GO molecular function analysis, *Ly75*-co-altered genes were enriched most significantly in the T cell receptor binding, phosphatidylinositol bis phosphate binding, and phosphatidylinositol-3,4- phosphate binding categorical terms (Figure 7b). Most enriched terms in the GO cellular component related to the integral component of the plasma membrane (Figure 7c). Gene enrichment analysis of *Ly75* and its co-altered gene set revealed that *Ly75* may be involved in the lymphocyte activation signaling pathways.

## 4. Discussion

Ly75, also known as DEC-205 and CD205, is one of the mannose receptor family, which consists of the *n*-terminal cysteine-rich domain, fibronectin type II repeat domain, and C-type carbohydrate recognition domains (CRDs). The mannose receptors are expressed primarily on the surfaces of macrophages and dendritic cells (DCs), therefore, the role of *Ly75* expression by DCs has been tremendously investigated; *Ly75* was found to play important roles in the phagocytosis of pathogens, endocytosis, and antigen presentation, ultimately leading to the initiation of immune responses [23]. Additionally, an in vivo study has demonstrated that targeting *Ly75* with antigen-coupled anti-*Ly75* monoclonal antibodies leads to antigen uptake, phagocytosis, and subsequent antigen presentation to T cells [52]. Interestingly, endocytosis by *Ly75* enables antigen presentation to CD8^+^ T cells and CD4^+^ T cells via major histocompatibility complex (MHC) class I molecules and MHC class II molecules [52]. Owing to the role of *Ly75* proteins that are expressed by DCs, various approaches using DC-based cancer immunotherapy have been developed [27]. Targeting *Ly75* using monoclonal antibodies against *LY75* has been an effective approach to induce both cellular and humoral immune responses. Importantly, this strategy has been applied to patients with malignant melanoma using patient-derived mature DCs. Consequently, it was found that targeting *Ly75* leads to effective antigen presentation, cytokine production, antigen-specific T cell stimulation, and T cell proliferation [53]. Additionally, in ovarian cancer, in vivo depletion of *Ly75*-positive DCs impairs tumor progression [29]. Therefore, these studies suggest that the expression of *Ly75* by DCs plays a crucial role in tumor immunity.

As such, although the effects of *Ly75* on anti-tumor responses and its role as a cancer immunotherapy have been investigated, the roles and clinical relevance of *Ly75* expression in melanoma have not yet been studied. Therefore, this study investigated the clinical relevance of *Ly75* expression on the survival rates of patients with melanoma by analyzing data in an integrated and systematic manner using various databases. As shown in Figure 1 and Figure 2, *Ly75* expression was significantly downregulated in skin cutaneous melanoma (SKCM), and its expression was positively correlated with the survival rates of patients with SKCM in multiple datasets (Figure 2 and Figure 3c), implicating that the downregulated expression of *Ly75* affects melanoma malignancy. Moreover, in SKCM, mRNA expression of *Ly75* was downregulated, while DNA methylation of the *Ly75* gene was upregulated, as compared to in normal tissues. DNA methylation is the most common epigenetics along with histone modifications and chromatin remodeling [54]. Abnormal methylation is evident in cancer development [42], and the hypermethylation of CpG islands is considered to play an important role in the progression of cancer by the inactivation of tumor suppressor genes [54,55]. A cohort study on melanoma and lung cancer shows that low methylation is related to decreased tumor immunity and poor clinical responses to immunotherapy [56]. This study demonstrated a significant negative association between lower mRNA expression and higher DNA methylation of the *Ly75* gene, suggesting that *Ly75* may be involved in melanoma progression. Particularly, DNA methylation of immune cells in a tumor microenvironment was recently reported to be specifically associated with poor survival for patients with melanoma [57]. As shown in Figure 4a, a significant negative correlation was found between tumor purity and *Ly75* expression, indicating that the *Ly75* gene may be expressed by infiltrated immune cells in the tumor microenvironment (TME) of SKCM [48].

*Ly75* expression is also reported to be detected in NK cells, macrophages, DCs, epithelial cells, and cancer cells [25]. Although NK cells play important roles in anti-tumor responses, the role of *Ly75* expression in NK cells has not yet been studied. In this study, *Ly75* expression was confirmed to be positively correlated to the infiltration of immune cells, such as NK cells, CD8^+^ T cells, and γδ T cells, which are known to act as anti-cancer effector cells (Figure 4). In fact, several studies have reported that NK cell infiltration in TME is markedly associated with good prognosis in various cancer types, such as colon cancer, gastric cancer, lung cancer, and melanoma [14,58,59,60]. NK cells are strong cytotoxic cells that are mediated through Fas-Fas ligand interactions and the release of cytotoxic molecules, such as granzymes and perforin [18]. NK cells, unlike CD8^+^ T cells, can kill cancer cells without pre-sensitized antigens. NK cell activation is determined by a balance between surface activation and inhibitory receptors, leading to spontaneous cytolytic activity against target cells [61]. Nevertheless, the activity and functions of NK cells are often inhibited in the TME [62,63]. The TME includes stromal cells and immune cells, which regulate tumor development. Resident immune cells in the TME comprise various lymphocytes, including T cells, B, cells, NK cells, macrophages, and DCs [64]. In the TME, certain types of immune cells, such as regulatory T cells and M2-macrophages, inhibit the activity of anti-tumor effector cells due to their immune-suppressive functions [63]. Although numerous lymphocytes infiltrate into the TME, the recruited lymphocytes also lose functionality due to immune-suppressive cells [65,66,67]. Therefore, it is tremendously important in anti-cancer therapy to maintain the activity of resident and recruited immune cells, as well as to increase the infiltration of immune cells into the TME. In this study, Figure 4 and Figure 5 show that *Ly75* expression exhibited a significant positive correlation with NK cell infiltration in SKCM. Moreover, the gene expression of activating receptors and cytolytic molecules was also increased by *Ly75* expression, which demonstrates that *Ly75* affects not only NK cell infiltration, but also NK cell cytolytic activity in SKCM. Therefore, this study supports using *Ly75* expression as a predictive biomarker for patient survival, NK cell infiltration, and NK cell activation in SKCM. Although these results suggest definite clinical associations of *Ly75* expression in SKCM, it has the limitation of analyzing only data available in the various databases. Therefore, further in vivo and in vitro studies that seek to logically support the results of these clinical associations are necessary.

In this study, to identify possible functions and biological processes associated with *Ly75* and its co-altered genes in SKCM, genes that were co-altered with *Ly75* were analyzed. Among the positively correlated genes, *protein tyrosine phosphatase receptor type C* (*Ptprc*) expression was most significantly co-altered with *Ly75* expression. PTPRC is the member of the protein tyrosine phosphatase (PTP) family, which regulates cell growth, differentiation, and oncogenic transformation. PTPRC, otherwise known as CD45, is a type I transmembrane protein, and is expressed in leukocytes, including T cells, B cells, and NK cells [68,69]. CD45 reportedly acts as a regulator for antigen receptor signaling of T cells and B cells, and CD45 activity is critical for efficient immune response. Particularly, in NK cells, CD45 expression is highly correlated with NK cell maturation [69,70]. CD45 expression has a significant association with KIRs and CD16 expression, which are markers of NK cell maturation [71]. Additionally, CD45 expression is higher in CD69^+^ NK cells than in CD69^-^ cells, suggesting that CD45 expression may be associated with NK cell cytolytic activity, as CD69 expression is highly associated with NK cytolytic activity [72]. Figure 6 showed that *Ly75* expression exhibits a significant positive correlation with *Ptprc* gene expression. This indicates that, as with CD45, *Ly75* expression may serve as a useful biomarker for predicting NK cell maturation and activation in SKCM. Next, to analyze the signaling pathways that are related with the 24 genes that are co-altered by *Ly75*, the Enrichr web tool was used, as shown in Figure 7. Using GO molecular function analysis, *Ly75*-co-altered genes were found to be enriched most highly in the T cell receptor binding, phosphatidylinositol bisphosphate binding, and phosphatidylinositol-3,4-phosphate binding categorical terms. Phosphorylation of phosphatidylinositol leads to form phosphatidylinositol phosphate (PIP), phosphatidylinositol bisphosphate (PIP_2_), and phosphatidylinositol trisphosphate (PIP_3_). Particularly, PIP_2_, including phosphatidylinositol 3,4-bisphosphate, phosphatidylinositol 3,5-bisphosphate, and phosphatidylinositol 4,5-bisphosphate, are phosphorylated from PIP by PI3K, and act as secondary messengers [73,74]. PIP_2_ plays a key role in immune systems, including neutrophil migration, T cell adhesion, phagocytosis, trafficking at the immune synapse of cytolytic effectors, and gene transcription in NK cells, suggesting the role of PIP_2_ in lymphocyte activation signaling pathways [75,76,77]. In NK cells, PIP_2_ plays a critical role in NK cell signaling and biological functions [78]. PI(4,5)P_2_ is reported to regulate cytolytic competency and immune synapse formation, and is then phosphorylated by PI3K, ultimately resulting in the formation of PI(3,4,5)P_3_. Subsequently, PI(3,4,5)P_3_ plays a role in cytokine production, chemotaxis, and NK homeostasis. After PI(3,4,5)P_3_ phosphorylation by SH2 domain-containing inositol-5-phosphatase (SHIP), PI(3,4)P_2_ is formed and regulates cytokine production, cytolytic competency, and NK homeostasis, indicating that PIP_2_ is critical for NK cell effector functions [78]. Moreover, PIP_2_ is required for the cytolytic activity of NK cells, specifically for the exocytosis process of cytolytic granules [77]. Therefore, this study conclusively suggests that *Ly75* and *Ly75*-co-altered genes are involved in NK cell functions.

## 5. Conclusions

In conclusion, this study used various publicly available databases and web tools to systematically analyze *Ly75* mRNA expression and its prognostic value in SKCM. Our analysis revealed that *Ly75* mRNA expression was significantly downregulated in SKCM compared to that in normal tissues. *Ly75* expression was positively correlated with clinical outcomes in patients with SKCM. Furthermore, *Ly75* expression exhibited a significant positive correlation with immune cell infiltration, especially that of NK cells. Moreover, the gene expression of NK cell activation markers was elevated along with *Ly75* expression, and *Ly75*-co-altered genes were involved in lymphocyte activation. Therefore, these results suggest that *Ly75* expression may increase patient survival rates via NK cell infiltration and activation in SKCM. Overall, our findings are the first to suggest the clinical relevance of *Ly75* expression in melanoma, as well as the potential of *Ly75* as a therapeutic target for melanoma.

## Figures and Tables

**Table 1:** Correlation between *Ly75* and markers of various immune cells in TIMER.

Description	Gene Markers	SKCM				COAD			
		None		Purity		None		Purity	
		Cor	*p*	Cor	*p*	Cor	*p*	Cor	*p*
T cell (general)	*CD3D*	0.776	***	0.642	***	0.000	0.994	0.019	0.704
	*CD3E*	0.769	***	0.634	***	0.034	0.467	0.072	0.150
	*CD2*	0.807	***	0.695	***	0.097	0.037	0.147	*
CD8^+^ T cell	*CD8A*	0.748	***	0.618	***	0.030	0.520	0.047	0.347
	*CD8B*	0.746	***	0.609	***	0.067	0.152	0.086	0.082
Th1	*T-bet (TBX21)*	0.765	***	0.635	***	0.047	0.317	0.099	0.047
	*STAT4*	0.790	***	0.694	***	0.124	*	0.143	0.004
	*STAT1*	0.666	***	0.588	***	0.244	***	0.268	***
	*IFNγ (IFNG)*	0.707	***	0.570	***	0.081	0.082	0.087	0.080
	*TNFα (TNF)*	0.687	***	0.540	***	0.073	0.121	0.102	0.039
Th2	*GATA3*	0.748	***	0.600	***	0.082	0.080	0.118	0.018
	*STAT6*	0.087	0.059	0.136	*	0.270	***	0.262	***
	*STAT5A*	0.272	***	0.327	***	0.216	***	0.227	***
	*IL13*	0.208	***	0.130	*	0.017	0.719	0.022	0.657
Tfh	*BCL6*	0.435	***	0.400	***	0.113	0.016	0.153	*
	*IL21*	0.583	***	0.482	***	0.104	0.026	0.102	0.040
Th17	*STAT3*	0.419	***	0.444	***	0.379	***	0.414	***
	*IL17A*	−0.048	0.294	−0.119	0.011	0.160	*	0.169	*
γδ T cell	*RORγt (RORC)*	0.400	***	0.280	***	0.158	*	0.145	*
	*CCR5*	0.816	***	0.722	***	0.148	*	0.188	**
	*CXCR6*	0.768	***	0.644	***	0.072	0.125	0.111	0.026
	*VNN2*	0.784	***	0.689	***	0.010	0.826	0.033	0.507
	*CD300A*	0.710	***	0.575	***	−0.048	0.304	−0.028	0.578
Treg	*FOXP3*	0.728	***	0.587	***	0.082	0.081	0.112	0.024
	*CCR8*	0.815	***	0.737	***	0.183	***	0.209	***
	*STAT5B*	0.403	***	0.509	***	0.455	***	0.467	***
	*TGF* *β (TGFB1)*	0.432	***	0.287	***	−0.037	0.425	−0.009	0.849
B cell	*CD19*	0.654	***	0.528	***	0.091	0.053	0.158	*
	*CD79A*	0.647	***	0.495	***	0.110	0.019	0.163	*
Monocyte	*CD86*	0.822	***	0.732	***	0.081	0.084	0.121	0.015
	*CD115 (CSF1R)*	0.753	***	0.648	***	0.059	0.210	0.085	0.088
TAM	*CCL2*	0.614	***	0.464	***	0.123	*	0.141	*
	*CD68*	0.422	***	0.231	***	0.078	0.094	0.113	0.023
	*IL10*	0.627	***	0.489	***	0.042	0.367	0.073	0.140
M1 Macrophage	*INOS (NOS2)*	0.100	0.029	0.118	0.012	−0.037	0.432	−0.033	0.511
	*IRF5*	0.641	***	0.478	***	0.218	***	0.227	0.000
	*COX2 (PTGS2)*	0.164	**	0.106	0.023	0.049	0.300	0.057	0.248
M2 Macrophage	*CD163*	0.663	***	0.550	***	0.086	0.066	0.110	0.026
	*VSIG4*	0.595	***	0.473	***	−0.019	0.683	−0.004	0.930
	*MS4A4A*	0.698	***	0.580	***	0.023	0.625	0.049	0.326
Neutrophils	*CD66b (CEACAM8)*	0.027	0.561	0.067	0.153	0.033	0.479	0.012	0.812
	*CD11b (ITGAM)*	0.671	***	0.570	***	−0.013	0.779	0.011	0.829
	*CCR7*	0.747	***	0.604	***	0.114	0.015	0.158	*
Natural killer cell	*KIR2DL1*	0.360	***	0.203	***	−0.161	*	−0.198	***
	*KIR2DL3*	0.490	***	0.302	***	−0.122	0.009	−0.129	*
	*KIR2DL4*	0.571	***	0.390	***	−0.100	0.032	−0.093	0.060
	*KIR3DL1*	0.461	***	0.283	***	−0.044	0.352	−0.053	0.290
	*KIR3DL2*	0.576	***	0.405	***	−0.048	0.301	−0.049	0.323
	*KIR3DL3*	0.138	*	0.067	0.155	−0.066	0.160	−0.058	0.241
	*KIR2DS4*	0.401	***	0.264	***	−0.085	0.068	−0.100	0.044
	*KLRK1 (NKG2D)*	0.722	***	0.597	***	0.026	0.575	0.047	0.344
	*NCR1 (NKp46)*	0.586	***	0.475	***	−0.046	0.330	−0.029	0.557
	*NCR2 (NKp44)*	0.302	***	0.247	***	−0.053	0.254	−0.049	0.327
	*NCR3 (NKp30)*	0.746	***	0.612	***	−0.030	0.523	0.017	0.733
Dendritic cell	*HLA-DPB1*	0.748	***	0.611	***	−0.055	0.240	−0.026	0.604
	*HLA-DQB1*	0.690	***	0.535	***	0.019	0.690	0.047	0.349
	*HLA-DRA*	0.785	***	0.668	***	0.033	0.481	0.062	0.211
	*HLA-DPA1*	0.734	***	0.606	***	0.073	0.118	0.114	0.022
	*BDCA1 (CD1C)*	0.671	***	0.535	***	0.150	*	0.186	**
	*BDCA4 (NRP1)*	0.505	***	0.456	***	0.151	*	0.206	***
	*CD11c (ITGAX)*	0.578	***	0.398	***	0.038	0.412	0.079	0.110

SKCM, skin cutaneous melanoma; COAD, colon adenocarcinoma; CD, cluster of differentiation; TAM, tumor-associated macrophage; Th, T helper cells; Tfh, Follicular helper T cells; γδ, Gamma Delta; Treg, regulatory T cells; Cor, R value of the Spearman’s correlation coefficient; None, correlation without adjustment. Purity, correlation adjusted by purity. * *p* < 0.01, ** *p* < 0.001, *** *p* < 0.0001.

**Table 2:** Correlation between *Ly75* and markers of anti-tumor effector cells in Gene Expression Profiling Analysis (GEPIA).

Cell Type	Gene Markers	SKCM				COAD			
		Tumor		Normal		Tumor		Normal	
		R	*p*	R	*p*	R	*p*	R	*p*
CD8^+^ T cells	*CD8A*	0.43	***	0.099	0.019	0.0028	0.96	0.56	***
	*CD8B*	0.45	***	0.14	**	0.062	0.31	0.6	***
NK cells	*KIR2DL1*	0.098	*	0.17	0.059	0.012	0.84	0.073	0.17
	*KIR2DL3*	0.17	**	0.038	0.038	0.024	0.69	0.24	***
	*KIR2DL4*	0.25	***	0.055	0.036	0.55	0.064	0.7	***
	*KIR3DL1*	0.31	***	0.067	0.12	0.0053	0.93	0.19	**
	*KIR3DL2*	0.42	***	0.043	0.42	0.014	0.81	0.5	***
	*KIR3DL3*	0.095	*	0.033	0.43	−0.04	0.51	0.16	*
	*KIR2DS4*	0.073	0.12	0.041	0.34	0.057	0.34	0.39	***
	*KLRK1 (NKG2D)*	0.48	***	0.18	***	0.0091	0.88	0.39	***
	*NCR1 (NKp46)*	0.21	***	0.087	0.039	0.047	0.44	0.16	*
	*NCR2 (NKp44)*	0.25	***	0.03	0.48	0.021	0.73	0.51	***
	*NCR3 (NKp30)*	0.62	***	0.2	***	−0.011	0.85	0.42	***
γδ T cell	*RORC (RORγt)*	0.05	0.28	0.016	0.7	0.06	0.32	0.81	***
	*CCR5*	0.51	***	0.14	**	0.092	0.13	−0.081	0.13
	*CXCR6*	0.48	***	0.17	***	0.1	0.083	0.65	***
	*VNN2*	0.65	***	−0.09	0.24	−0.12	0.046	0.029	0.59
	*CD300A*	0.51	***	0.12	*	−0.072	0.23	0.54	***

NK, natural killer; *** *p* < 0.0001, ** *p* < 0.001, * *p* < 0.01.

**Table 3:** Correlation between *Ly75* and NK activation signature genes using TIMER.

	Gene Markers	SKCM		COAD	
		R	*p*	R	*p*
Activation receptors	*KLRK1*	*0.722*	***	*0.026*	*0.575*
	*NCR1*	*0.586*	***	*−0.046*	*0.330*
	*NCR2*	*0.302*	***	*−0.053*	*0.254*
	*NCR3*	*0.746*	***	*−0.030*	*0.523*
FAS & FASL	*FAS*	*0.529*	***	*0.032*	*0.494*
	*FASLG*	*0.752*	***	*0.078*	*0.097*
Cytolytic molecules	*GZMA*	*0.753*	***	*0.006*	*0.891*
	*GZMB*	*0.670*	***	*0.096*	*0.040*
	*PRF1*	*0.697*	***	*0.004*	*0.928*

*** *p* < 0.0001.

## Supplementary Materials

The following are available online at https://www.mdpi.com/2077-0383/9/5/1383/s1, Table S1: Numbers of TCGA tumor and normal samples and their paired GTEx samples in GEPIA. Table S2: COX regression results for *Ly75* with TCGA data in various types of cancers by OncoLnc (http://www.oncolnc.org/), Table S3: Correlation analysis between *Ly75* and infiltrated immune cells in TIMER.
