# Supplementary material for: Systematic Multiomic Analysis of Ly75 Gene Expression and Its Prognostic Value through the Infiltration of Natural Killer (NK) Cells in Skin Cutaneous Melanoma
Source: J Clin Med. 2020 May 8;9(5):1383. doi: 10.3390/jcm9051383 (PMC7291273; doi:10.3390/jcm9051383)
Supplement: Supplementary file 1 [file jcm-09-01383-s001.pdf]

Supplementary Table S1. Numbers of TCGA tumor and normal samples and their paired GTEx samples in GEPIA.

TCGA type	Detail	Tumor sample	Normal sample	GTEx tissue source	GTEx sample
ACC	Adrenocortical carcinoma	77	-	Adrenal Gland	128
BLCA	Bladder Urothelial Carcinoma	404	19	Bladder	9
BRCA	Breast invasive carcinoma	1085	112	Breast	179
CESC	Cervical squamous cell carcinoma and endocervical adenocarcinoma	306	3	Cervix Uteri	10
CHOL	Cholangio carcinoma	36	9	-	-
COAD	Colon adenocarcinoma	275	41	Colon	308
DLBC	Lymphoid Neoplasm Diffuse Large B-cell Lymphoma	47	-	Blood	337
ESCA	Esophageal carcinoma	182	13	Esophagus	273
GBM	Glioblastoma multiforme	163	-	Brain	207
HNSC	Head and Neck squamous cell carcinoma	519	44	-	-
KICH	Kidney Chromophobe	66	25	Kidney	28
KIRC	Kidney renal clear cell carcinoma	523	72	Kidney	28
KIRP	Kidney renal papillary cell carcinoma	286	32	Kidney	28
LAML	Acute Myeloid Leukemia	173	-	Bone Marrow	70
LGG	Brain Lower Grade Glioma	518	-	Brain	207
LIHC	Liver hepatocellular carcinoma	369	50	Liver	110
LUAD	Lung adenocarcinoma	483	59	Lung	288
LUSC	Lung squamous cell carcinoma	486	50	Lung	288
MESO	Mesothelioma	87	-	-	-
OV	Ovarian serous cystadenocarcinoma	426	-	Ovary	88
PAAD	Pancreatic adenocarcinoma	179	4	Pancreas	167
PCPG	Pheochromocytoma and Paraganglioma	182	3	-	-
PRAD	Prostate adenocarcinoma	492	52	Prostate	100
READ	Rectum adenocarcinoma	92	10	Colon	308
SARC	Sarcoma	262	2	-	-
SKCM	Skin Cutaneous Melanoma	461	1	Skin	557
STAD	Stomach adenocarcinoma	408	36	Stomach	175
TGCT	Testicular Germ Cell Tumors	137	-	Testis	165
THCA	Thyroid carcinoma	512	59	Thyroid	278
THYM	Thymoma	118	2	Blood	337
UCEC	Uterine Corpus Endometrial Carcinoma	174	13	Uterus	78
UCS	Uterine Carcinosarcoma	57	-	Uterus	78
UVM	Uveal Melanoma	79	-	-	-

Supplementary Table S2. COX regression results for *Ly75* with TCGA data in various types of cancers by OncoLnc (http://www.oncolnc.org/). The data with p-value < 0.01 are marked with red.

Cancer	Cox Coefficient	p-value	FDR-Corrected	Median Expression	Mean Expression
BLCA	-0.118	0.120	0.348	590.62	747.37
BRCA	-0.085	0.320	0.683	409.03	564.26
CESC	-0.103	0.460	0.769	724.40	818.15
COAD	0.017	0.870	0.961	1671.78	1793.53
ESCA	-0.093	0.490	0.979	1021	1265.59
GBM	0.168	0.093	0.788	145.24	166.62
HNSC	-0.053	0.460	0.759	487.58	674.91
KIRC	-0.102	0.200	0.317	491.15	645.67
KIRP	0.217	0.130	0.297	126.77	323.32
LAML	-0.100	0.370	0.736	2970.73	3064.58
LGG	0.407	2.40×10^{-5}	1.59×10^{-4}	83.59	111.21
LIHC	-0.123	0.180	0.435	48.66	92.31
LUAD	-0.174	0.034	0.163	609.01	762.9
LUSC	0.057	0.430	0.841	453.33	639.24
OV	-0.001	0.990	0.994	594.17	667.78
PAAD	0.288	0.014	0.090	1251.64	1337.29
READ	-0.252	0.260	0.943	1869.78	2080.17
SARC	-0.382	6.80×10^{-4}	2.75×10^{-2}	110.07	186.48
SKCM	-0.239	2.90×10^{-4}	5.88×10^{-3}	83.34	219.56
STAD	-0.030	0.700	0.906	1286.64	1420.02
UCEC	-0.004	0.970	0.995	452.12	634.26

Supplementary Table S3. Correlation analysis between *Ly75* and infiltrated immune cells in TIMER

Immune Cells	SKCM		COAD	
	partial.cor	*p*	partial.cor	*p*
Purity	-0.565	4.88×10^{-40}	0.073855	0.137
B Cell	0.343	8.23×10^{-14}	0.172018	0.0005
$CD8^+$ T Cell	0.613	1.24×10^{-46}	0.157013	0.0015
$CD4^+$ T Cell	0.426	3.87×10^{-21}	0.319722	5.26×10^{-11}
Macrophage	0.438	1.13×10^{-22}	0.235994	1.61×10^{-6}
Neutrophil	0.711	7.79×10^{-71}	0.200845	5.11×10^{-5}
Dendritic Cell	0.653	1.36×10^{-55}	0.212197	1.78×10^{-5}
